# Imperfection works: Survival, transmission and persistence in the system of Heliothis virescens ascovirus 3h (HvAV-3h), *Microplitis similis* and *Spodoptera exigua*

**DOI:** 10.1038/srep21296

**Published:** 2016-02-16

**Authors:** Shun-Ji Li, Richard J. Hopkins, Yi-Pei Zhao, Yun-Xuan Zhang, Jue Hu, Xu-Yang Chen, Zhi Xu, Guo-Hua Huang

**Affiliations:** 1 Hunan Provincial Key Laboratory for Biology and Control of Plant Diseases and Insect Pests, Hunan Agricultural University, Changsha, Hunan 410128, China; 2 Institute of Virology, Hunan Agricultural University, Changsha, Hunan 410128, China; 3 Natural Resources Institute, University of Greenwich, Kent ME4 4TB, UK

## Abstract

Ascoviruses are insect-specific large DNA viruses that mainly infect noctuid larvae, and are transmitted by parasitoids in the fields. Heliothis virescens ascovirus 3h (HvAV-3h) has been recently isolated from *Spodoptera exigua*, without parasitoid vector identified previously. Here we report that *Microplitis similis*, a solitary endoparasitoid wasp, could transmit HvAV-3h between *S. exigua* larvae in the laboratory. When the female parasitoid wasp acquired the virus and served as a vector, the period of virion viability on the ovipositor was 4.1 ± 1.4 days. Infected host larvae were still acceptable for egg laying by parasitoids, and the parasitoids thereafter transmitted virus to healthy hosts. Virus acquisition occurred only from donor hosts between 3 and 9 days post infection. The peak of virus acquisition (80.9 ± 6.3%) was found when *M. similis* wasps oviposited in larvae that had been inoculated with the virus 7 days previously. When virus infection of the host took place during the life cycle of the parasitoid wasp, it caused 1- to 4-day-old immature parasitoids death in the host, whilst a small proportion of 5- to 6-day-old and the majority of 7-day-old parasitoids larvae survived from the virus-infected hosts. Viral contamination did not reduce the life span or fecundity of female *M. similis*.

The beet armyworm, *Spodoptera exigua* (Hüber) (Lepidoptera: Noctuidae), is a widely distributed omnivorous pest, considered economically important to commercial growers of cotton and most plants in the Brassicaceae^1^. To effectively control *S. exigua*, chemical pesticides are applied extensively, with consequent serious environmental issues^2^ and the destruction of natural enemies^3^,^4^. Owing to the susceptibility of *S. exigua* to many pathogens, i.e., nucleopolyhedrovirus (NPV), over the past forty years, such insect viruses have been used as biological control agents to suppress the pest^5^ and to reduce the use of chemical pesticides. Apart from baculoviruses, ascoviruses, belonging to the family *Ascoviridae*, are also important pathogens of *S. exigua*. Ascoviruses were discovered in the late 1970s and named in the early 1980s^6^,^7^,^8^, five species in a single genus are currently recognized; they are *Spodoptera frugiperda ascovirus 1a*, *Tricoplusia ni ascovirus 2a*, *Heliothis virescens ascovirus 3a*, *Diadromus pulchellus 4a* and *Tricoplusia ni ascovirus 6a*^9^,^10^. However, due to the unique pathology of ascoviruses, and other large double-stranded DNA viruses^11^,^12^, little effort has been extended to investigate the possibility of using an ascovirus for pest control.

One of the difficulties in studying ascoviruses involves the lack of apparent infection symptoms in field studies. Notwithstanding, the stunting of growth and a yellowish color of infected larvae sometimes aids the diagnosis of the disease under laboratory conditions^13^,^14^. On the other hand, ascoviruses, with the low gross infection rate, from 0.26% to 50%^8^,^15^,^16^, probably have long been regarded as insignificant for pest control. To some extent, this low infection rate in the field may be attributed to the mode of transmission which relies much on the parasitoid wasp population^16^,^17^,^18^.

Unlike other pathogens that have been successfully industrially produced for biocontrol agents, ascoviruses are rarely transmitted *per os*^15^,^19^; however, they can be efficiently transmitted by hymenopteran endoparasitoids, which introduce virus particles into susceptible hosts during the act of oviposition^17^,^20^,^21^. To date, it is known that among hymenopteran species, some of the Braconidae and Ichneunmonidae species can efficiently transmit ascoviruses during the process of oviposition^21^,^22^. In France, *Diadromus pulchellus 4a* was isolated from its vector host, *Diadromus pulchellus*^20^. In the US, where *Spodoptera frugiperda ascovirus* (SfAV), *Tricoplusia ni ascovirus* (TnAV) and *Heliothis virescens ascovirus* (HvAV) were isolated, *Cotesia marginiventris* served as a vector to transmit Sf 82–126 (belongs to SfAV)^22^. *Cardiochiles nigriceps, Microplitis croceipes* as well as *Campoletis sonorensis* were able to transmit HV99-14 (belongs to HvAV) at a notable high transmission rate^21^.

Although many parasitoid species have been studied in terms of their ascovirus transmission capabilities, it remains necessary to elucidate the diverse relationship between ascoviruses and many parasitoids vectors. Here, we report Heliothis virescens ascovirus 3h (HvAV-3h), belonging to the HvAV complex^16^,^23^, is transmissible among *S. exigua* hosts via *Microplitis similis* Lyle (Hymenoptera: Braconidae). *Microplitis similis* is a koinobiont parasitoid wasp that spends its immature stage in the host cavity and allows the host to continue feeding and moving until a cocoon forms^24^. It is known that parasitoid wasps can mediate host development^25^, and serve as a model of optimal resource allocation to fitness, for example, host stadium and developmental time^26^,^27^. Elucidating the parasitoid-wasp, which mediates viral transmission, is important for not only understanding ascoviruses biology, but also for host – parasitoid wasp – pathogen system coevolution. Here, we determined the transmission rates under different viral propagating stages, the virion viability on the ovipositor and the effects of HvAV-3h infection on *M. similis* development to understand the putative HvAV-3h transmission mechanism.

## Results

### *Microplitis similis* serves as vector

#### Virus diminishes gradually on the ovipositor

After a single sting on an infected host (donor host), *M. similis* could deliver the virus to recipient hosts, which could be confirmed by the milky-white color in the recipient *S. exigua* hemolymph. Given this evidence on the successful viral transmission via *M. similis*, in the system of HvAV-3h, *S. exigua* and *M. similis* itself, *M. similis* served as a vector, transmitting virus among *S. exigua* individuals. However, the recipient hosts included both virus infected (VI, 51.00 ± 6.63%) and un-infected individuals. Un-infected individuals included both parasitized (cocoon-producer, CP, 9.54 ± 2.31%) and non-parasitized individuals (moth formation, MF, 41.00 ± 4.54%) (*F*_2, 87_ = 19.94, *P* < 0.05).

**Figure 1:**
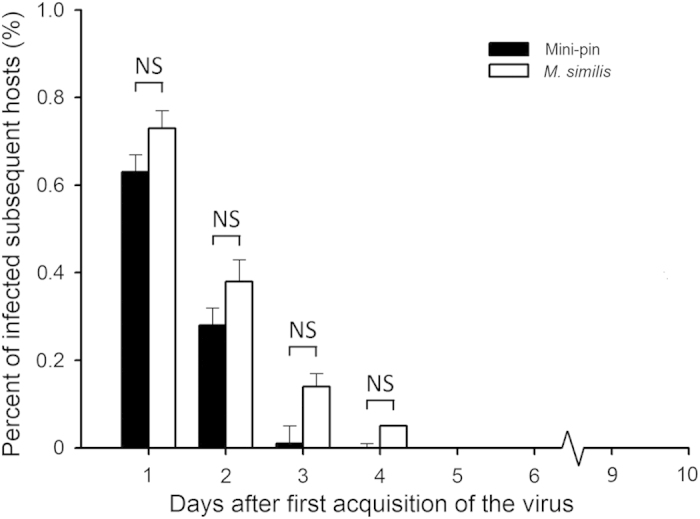
Transmission of HvAV-3h with contaminated ovipositor and mini-pin. Rates of transmission was indicated by contaminated ovipositors in day 1, day 2, …, day 10 after attacking the first infected-hosts; and by contaminated mini-pins in day 1, day 2, …, day 10 after dipping into HvAV-3h solution without drying and secondary dipping within time intervals. NS, not significantly different.

If a virus-contaminated ovipositor of the vector transmitted the virus mechanically, then the virion on the vector’s ovipositor would diminish gradually as it stung a number of subsequent hosts. To test this hypothesis, we allowed 30 vectors that had previously stung donor hosts (thus they became viruliferous vectors) to individually parasitize a quota of 5 host larvae each day until death, the results showed that the average time which the vector retained the virus was 4.10 ± 1.44 days (Fig. 1). The first day had seen the largest percent of VI, in which the viruliferous vectors were allowed to parasitize the first quota of 5 subsequent hosts shortly after they stung infected hosts. The percentage of VI dropped significantly from day 2 to day 3 (day 2: 38.22 ± 5.71%; day 3: 14.28 ± 3.20%; Fig. 1). At day 4, the percentage of infected subsequent hosts was the lowest (3.27 ± 1.83%; Fig. 1) in which the viruliferous vectors had already been allowed to parasitize for 5 days (the first day of parasitism were on donor hosts; Fig. 1). Occasionally, very few of the viruliferous vectors could successfully deliver the virus on day 5 (6.36 ± 3.26%; Fig. 1) or 6 (4 out of 30 on observation).

Mini-pin is typically used to simulate the mechanical transmission of virus through the ovipositor in ascovirus research^8^,^12^,^19^,^28^. In this study, we had each mini-pin dipped into the virus suspension and inoculate 5 hosts per day for a total of 10 testing days. The percentage of infected subsequent hosts was highest on the first day, and then progressively dropped on the second day and the third day; until by the fourth day only a negligible percentage of hosts became infected. No significant difference was found between the percent of infected hosts from the mini-pin compared to the real vector (Day 1: *F*_1,9_ = 1.49, *P* = 0.22; Day 2: *F*_1,9_ = 0.13, *P* = 0.71; Day 3, *F*_1,9_ = 0.52, *P* = 0.47; Day 4: *F*_1,9_ = 0.47, *P* = 0.50; Fig. 1). These results showed evidence that HvAV-3h was transmitted via *M. similis* through a mechanical process. The virus gradually had been wiped off after a series of stings on recipient hosts, the same as the process that virus was brought into the hosts by using mini-pins.

#### Ovipositor acts differently resulting in infected and uninfected recipient hosts

The overall infection rate of recipient hosts demonstrated a downward trend with the observational period, showing no significant difference with the mini-pin trial. It is interesting to note that we observed a low proportion of infected hosts in the fourth day to sixth day, when the virus particles sticking to the ovipositor of the vector was supposed to be reduced by serially penetrating on recipient hosts (data not shown). The success of mechanically transmission also depended on the depth to which the ovipositor penetrated the recipient host. The deeper the penetration on the donor host, the more virus particles the vector acquired. When the vector oviposits in recipient hosts, the number of virus particles adhering to the ovipositor will reduce progressively. A light penetration of a recipient, where the tip of the ovipositor did not release virus particles acquired from the donor host, the second recipient host may not be infected. Deeper penetrations would be more likely to result in a recipient host becoming infected. In our result, infected recipient hosts and uninfected recipient hosts were within the same test period (daily), supporting the penetration differences of the vector as one source of the variation in the success infecting recipient hosts (Fig. 1).

#### A competent vector contributes transmission rate with high concentration from donor hosts

**Figure 2:**
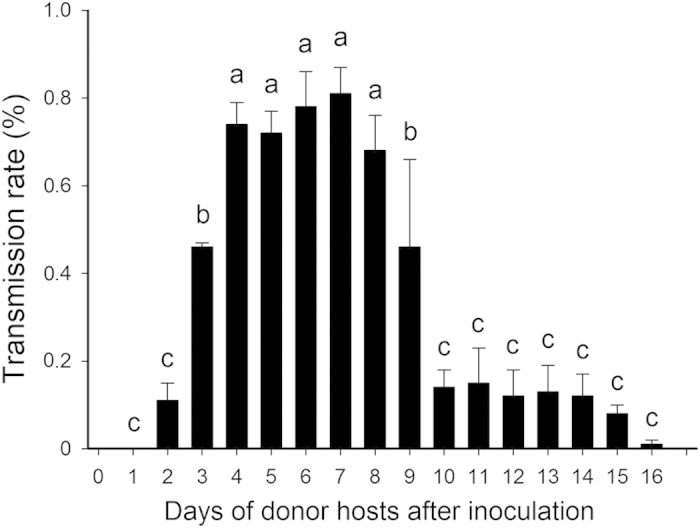
Rates of viral transmission from hosts in different disease developmental stage. Hosts provided for viral transmission were manually inoculated with HvAV-3h and then exposed to female parasitoids for 1, 2, …, and 16 days post-inoculation. There were totally 10 female parasitoids and about 100 subsequent hosts in each time.

Previous studies have indicated that the concentration of ascovirus in the hemolymph of a host presented an upward trend peaking 7 to 8 days post-infection (DPI) according to species^19^, and then a gradual downward trend until death of the host. We therefore examined the viral transmission rate the vector contributed to changes according to the accumulating and diminishing of vesicles within the donor hosts. We found a positive correlation between the progressing propagation of vesicles in the donor host hemocoel and the percentage of infected subsequent hosts (viral transmission rate) within the first 8 days (Fig. 2). The vector parasitoids that stung donor hosts 1 DPI caused negligible viral infection rates through oviposition in subsequent hosts. Transmission of virus could be identified at a low rate (11.08 ± 4.03%) following ovipositing on 2 DPI donor hosts and then subsequent hosts, but the data of 1 DPI and 2 DPI did not differ significantly (Fig. 2). 3 DPI to 7 DPI donor hosts were linked to vectors with a notably high viral transmission rate. The transmission rate regressed from 8 DPI to 10 DPI donor hosts (Fig. 2). Infection rates from 10 DPI to 16 DPI donor hosts were low, and did not significantly differ from each other (P < 0.05). It is important to note that conducting the viral transmission experiments on 9 DPI donor hosts and above was difficult. The vector parasitoids seldom laid eggs in these hosts or they had difficulty in searching for these hosts, and thus their subsequently parasitized host did not show viral infection.

Transmission rate from 7 DPI donor hosts was the highest (80.85 ± 6.32%). Nevertheless, after a single oviposition in a virus-infected host, each of the vector parasitoids did not deliver the virus to every subsequent host. The fate of subsequent hosts included virus-infection (51.00 ± 6.63%), cocoon-production (9.54 ± 2.31%) and moth-formation (41.00 ± 4.54%) (*F*_2, 87_ = 19.94, *P* < 0.05).

#### Host-marking behavior cuts down but does not block viral transmission rate

A vector parasitoid wasp with a contaminated ovipositor introduces its egg into the host cavity along with some viral particles so that the recipient host cavity contains undeveloped immature parasitoids. The newly infected host becomes a donor host and a second vector, which parasitizes this host, become another viruliferous vector. Solitary parasitoid wasps like *M. similis* have been proven to demonstrate some host-marking behavior, in which they hesitate to oviposit in a previously parasitized host. In ascovirus studies, host-marking behavior may block ascovirus transmission since this virus could only spread through parasitoid oviposition. To better understand this, we had the donor hosts once parasitized by viruliferous vectors, and after 7 days (when the concentration of virus in host hemolymph peaks), we had them exposed to another virus-free parasitoid and then we made observation on the latter vectors in terms of their viral transmission abilities. The result showed that all these vectors become viruliferous (*N* = 6), indicating that donor host acquiring the virus originally from a viruliferous vector could be used as a virus resource. However, these vectors could only contributed to 10.76 ± 0.49% transmission rate, dramatically lower than transmission from manual-inoculated donor hosts (Fig. 2). Although we did not prove whether *M. similis* carry out strict host-marking behavior, we did reveal a significant reduction in viral transmission rate from parasitized donor hosts.

### Effects of HvAV-3h on the life cycle of *M. similis*

#### Virus kills young but not older immature *M. similis*

**Figure 3:**
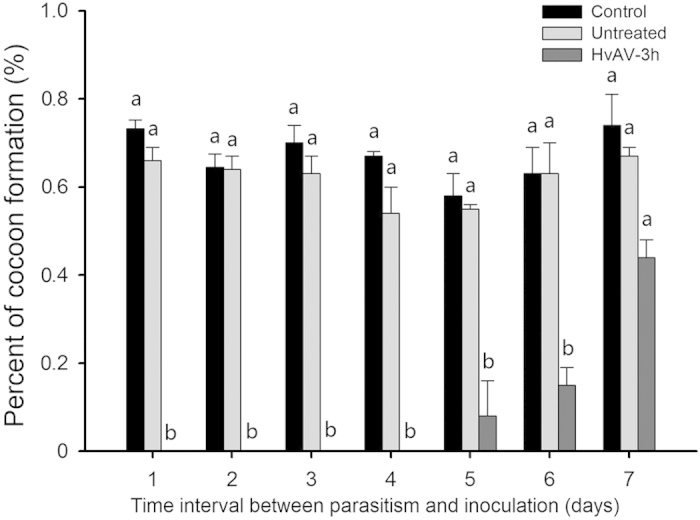
Survival of parasitoids in the host. Hosts were manually inoculated with HvAV-3h at 1, 2, …, 6 and 7 days post-parasitism. Blank control larvae were not inoculated, and control larvae were inoculated with hemolymph from healthy larvae. Here, the day when the hosts were parasitized was defined as day 1 rather than day 0.

This experiment was designed to understand the fate of immature parasitoids of different ages when their host becomes virus infected. When the hosts were parasitized 1 day prior to virus inoculation (1 day interval, 1 DI), they did not produce cocoons. After 9 days, we dissected these hosts and we found dead 1-2-day-old immature parasitoids in the host cavities in the host’s milky-white hemolymph. When the hosts were parasitized 2, 3 or 4 days prior to virus inoculation, they also failed to produce cocoons (Fig. 3). However, when a cluster of hosts were parasitized 5 days prior to being inoculated with the virus, a small proportion of them could produce cocoons (Fig. 3); for 6 and 7 days prior to inoculation, the percent of cocoon production by the hosts rose; at 7 DI, the data showed no significant differences to untreated control (Fig. 3). This indicates that as the age of the parasitoid immature increased at the time of virus infection, the virus posed less threat to the parasitoid completing development. Hosts from treated groups were dissected to find out whether their cavity contained parasitoids immature at the time when parasitoids egression in their untreated control group was about to begin. At 1 DI, the immature parasitoids were found with possible 1-instar in the host cavity, and the hosts were observed to have milky-white hemolymph. The remaining 5 DI, 6 DI and 7 DI hosts that did not produce cocoons, on the other hand, lived for several days showing an elongated larval stage before death, which is a typical symptom of ascovirus infection. Dissection result showed that immature parasitoids died within these host cavities at an advanced larval stage.

#### Virus does not affect *M. similis* adult behavior

A small proportion of parasitoids were able to develop in hosts inoculated with the virus 5 DPI to 7 DPI (referred to as Type I). After their emergence, female and male parasitoid wasps were paired and the mated females were allowed to parasitize hosts. The number of eggs laid showed no significant difference between groups (Table 1). Cocoons that had developed from eggs laid by viruliferous vectors (in the first experiment) were collected and kept until they emerged, and the subsequent adults were referred to as Type II. After mating within group, the females were allowed to lay eggs in the hosts. The fecundity of these females was not significantly different from each group (*F*_2,93_ = 0.93, *P* = 0.42; Table 1) and nor was their life span different from other types of vectors (*F*_2,93_ = 1.85, *P* = 0.14; Table 1). Type I and Type II vectors investigated did not deliver virus to subsequent hosts (data not shown).

## Discussion

Previous studies indicated a putative mechanical transmission of lepidopteran ascoviruses, and here, our results give more details on how the system works. We found a positive correlation between vesicle concentration in the host hemolymph and viral transmission rate on subsequent hosts. Additionally, there was a gradual diminishing of virus transmission over time, almost certainly linked to mechanical transmission of the virus and an associated progressive reduction in the number of virus particles on the ovipositor. Donor hosts through parasitization acted as a virus resource regardless of host-marking behavior. In addition, we demonstrate that a proportion of the older stages of *M. similis* immature (5–7 days old) could survive the infection of their host, and that HvAV-3h did not affect the reproduction and life cycle of the vector, nor show any vertical transmission in the vector’s population.

These results demonstrate how the system of host, vector and virus interact with three imperfection aspects. Firstly, HvAV-3h is spread by the oviposition behavior of the vector and host-marking behavior does not block this transmission; however, virus gradually diminishes on the ovipositor of the vectors over about 4 days after acquiring the virus and thus can be linked to both infected hosts and cocoon-producing hosts, the latter sustaining the population of the vector. Secondly, the virus could kill the host and 1–4-day-old immature parasitoids but not 5–7-day-old, the latter maintaining vector reproduction. Thirdly, the virus does not biologically affect the life span and oviposition behavior of female parasitoids. Whilst mechanical transmission of the virus can be regarded as relatively ineffective, paradoxically, such imperfection allows the system to function. Through the mechanisms listed above, the vector, even if it deposits on an infected host, can still reproduce effectively.

Our study reveals that under laboratory conditions, virus-free vector did oviposit in a donor host containing conspecific larvae. It is proven that the oviposition behavior of parasitoid wasps is often modified by the presence of conspecifics brood^29^. However, there was infection in subsequent hosts originating from parasitized virus-donor hosts although at a significant lower rate compared with donor hosts that were infected by manual inoculation. The feasibility of viral acquisition by a newly introduced vector from a donor host, parasitized by a viruliferous vector, may be an artefact, due to the absence of an alternative host resource in a narrow test tube environment. When unparasitized hosts are not available, parasitizing an already parasitized host increases the fitness of the female^30^. Thus, the situation becomes complex since the feeding environment of the host provides a more complex context for chemical signal cues for both prey and predator species^31^. Given this, although our model has been proven to work, field studies must be added to confirm these processes in a natural environment.

That HvAV-3h has a mechanically transmission was indicated by our finding that the infection rate of subsequent hosts fell dramatically after three days. The variation in the depth of penetration of the ovipositor may contribute to the virus-carrying vector producing both infected (VI) and uninfected subsequent (CP) hosts. As was suggested by Tillman *et al.*, the virions may adhered to the cuticle of the ovipositor^21^, and our results are in alignment with that. Mechanical transmission, though, has been characterized more in plant virus transmission mediated by insect vectors as semipersistent transmission, or non-circulative transmission, in which the virus is retained in the insect’s stylet or foregut^32^. The answer to the question that which insect species can serve as the respective vector of a virus lies in the interaction between the virus encoding helper component and the specific insect’s binding sites^33^. In ascovirus, whether this interaction exists is not clear since there are little data analyzing the molecular interaction between ascoviruses and either their vectors or hosts.

When the parasitoids immature competes with the virus for the same host, the costs to the former should be severe^34^. However, costs may be mitigated if the virus enters the host 5 to 7 days later than a parasitoid egg, and a few parasitoids would then complete successful development. Therefore, HvAV-3h, like other reported lepidopteran ascoviruses so far, is detrimental to the development of parasitoids^15^,^21^. DpAV-4a, serves as an exception, placing no pathogenic effects on its vector parasitoids wasp, *Diadromus pulchellus*^20^. It integrates its DNA into its vector’s genome and involves vertical transmission in the vector’s population^35^. On the other hand, lepidopteran ascoviruses, such as HvAV complex, showed little evidence that they could integrate into the genome of the vector^21^,^22^. Through phylogenetic analysis, DpAV-4a should be considered another lineage rather than other lepidopteran ascoviruses^36^. Furthermore, the process of integrating DNA into the host genome is very like the biology of polydnaviruses^37^, providing the hypothesis that polydnaviruses share a common ancestor with ascoviruses^38^,^39^.

HvAV-3h is not likely to remain active in the *M. similis* population through vertical transmission, suggested by the facts that three types of vector associated with HvAV-3h in this study exhibited little difference in life span and reproductive ability; nor did the Type I and Type II vectors deliver virus to their progeny.

**Figure 4:**
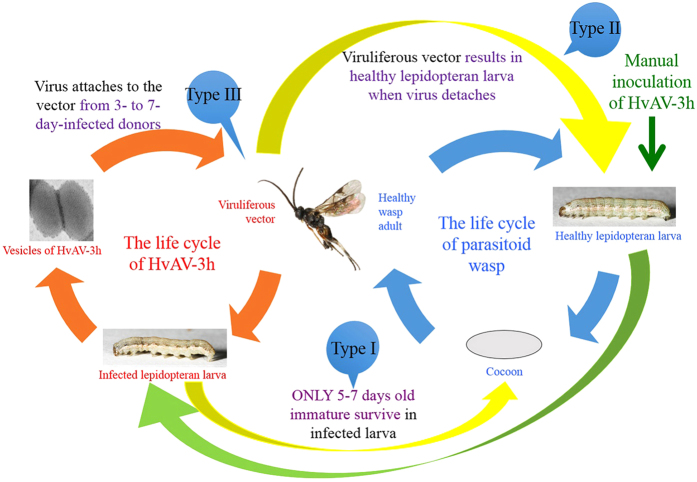
The system of parasitoid-host-virus contains two cycles: the life cycle of HvAV-3h and the life cycle of parasitoid wasp. The elements in both cycles are dependent to the other elements. Any changes of nature of any elements in the cycles result in disruption of the cycles. *Microplitis similis* plays the most important role in both cycles, providing overlapping for both cycles. In the life cycle of parasitoid wasp (with blue arrows), the parasitoid wasp here is referred to healthy parasitoid wasp, which parasitizes lepidopteran larva, and the latter produces cocoon of parasitoid wasp, emerging as parasitoid wasp adult. In the life cycle of HvAV-3h (with blue orange arrows), the parasitoid wasp here is referred to viruliferous vector. The virus (from donor) attaches to parasitoid wasp’s (becomes viruliferous vector) ovipositor, and be introduced into lepidopteran larva’s cavity through parasitism of the viruliferous vector. The lepidopteran larva then becomes infected (donor), and provides virus for other parasitoid wasp since host-marking does not block viral transmission. The system containing both cycles is supposed to keep balance for itself by two scenarios (with yellow arrows): (**a**) viruliferous vector parasitism results in both infected and uninfected larvae; (**b**) larva parasitized by healthy parasitoid wasp then by viruliferous vector can produce healthy parasitoid wasp cocoon. The green arrow represents the experimental setting in our study. Purple words indicate the experimental data we got in this study. (All the photos taken by ourselves).

In summary, our results reveal major components of a model that includes two cycles (Fig. 4). The virus-vector-host cycle of HvAV-3h is overlapping with the life cycle of *M. similis* through the oviposition behavior of the *M. similis* female. For HvAV-3h to be effective, the behavior pattern of *M. similis* is a prerequisite so that the vector could continuously deliver the virus to fresh hosts. For this to be sustainable, it is also a condition that the virus does not extensively disrupt the life cycle of *M. similis*. This is where the importance of the imperfection lies: we have proven in the study that HvAV-3h did not kill all immature *M. similis*, and viruliferous *M. similis* would not always result in the death of the host through viral transmission. In addition, the mechanical transmission of HvAV-3h allows females to lay eggs in uninfected hosts and even infected hosts, improving her reproductive success. These points preserve the reproduction of *M. similis* and allow the integrity of the two cycles of the system. Future work must lead to an understanding of the transmission mechanism of HvAV-3h from an ecological view in favor of biological control. Thus, we must understand the trade-off of parasitoid reproduction in the field, versus virus vectoring of HvAV-3h and how these two overlapping systems influence the damage of the pest. Further, more details must be added through research such as the host selection and discrimination of the vector, as well as the host-marking behavior of the vector in the field.

## Methods

### Maintenance of insects and virus culture

All insects were reared in a chamber with a controlled temperature at 27 ± 2 °C, RH at 70 ± 10%, 14L:10D. *Spodoptera exigua* were from colonies maintained in our laboratory^23^. *Microplitis similis* was collected in a cotton field near Hunan Agricultural University, Changsha, Hunan, China, and reared under laboratory condition^24^.

The genders of newly emerged parasitoids adults were determined by recognizing the presence of the ovipositor under the microscope. Males and females were fed on 30% honey solution. Each pair was provided with second- or third-instar *S. exigua* larvae for propagation.

The virus used, HvAV-3h, was from a stock maintained by our laboratory (1.1 × 10^11^ virion copies/ml)^23^.

### Acquisition and transmission of HvAV-3h by *M. similis* female

All experiments were conducted in a control temperature and humidity environment (27 ± 2 °C, humility 70 ± 10%, L14:D10). Determination of viruliferous vectors was conducted following Tillman *et al.*^21^. If not otherwise stated, all larvae once exposed to the parasitoids were transferred to separate test tubes (2 × 10 cm) containing artificial diet for daily observation.

### Vesicle viable on ovipositor and mini-pin

Each 2-d-old viruliferous female wasp was provided with 5 second- or third-instar *S. exigua* larvae each day until death. The larvae parasitized were monitored daily to see whether they were VI or CP. A total of 30 vectors were included as repeats. Thirty 2-d-old vectors without virus contamination were used as a control.

A mini-pin contaminated with HvAV-3h was used to stab 5 third-instar larva for the one-day treatment, and another 5 larvae at the second day, and so forth. At an interval of 24 hours, the pin was kept exposed without drying or dipping into the virus solution again. This experiment was conducted for 10 consecutive days, with 10 mini-pins as repeats. The inoculated larvae were reared separately to observe whether they were IV.

### Transmission rates of HvAV-3h from donor hosts

Several 2-d-old females were allowed to parasitize VI larvae (1, 2, 3, …, 14 and 15 days post-inoculation, DPI; 10 replicates for each DPI). As soon as oviposition was observed, each female wasp was allowed to parasitize 10 larvae for 24 hours in a tube. These 10 larvae were transferred to test tubes and reared separately to check for VI or CP.

To test if there was a difference on virus transmission rate from different resource of donor host (manually injected virus and parasitized injected virus), totally 10 females were introduced into tubes separately containing donor hosts which had been parasitized by a viruliferous female. After 24 hours, the 10 females were allowed to parasitize 10 subsequent hosts. The infection of the subsequent hosts was compared to those from manually-injected-virus-hosts.

### *Microplitis similis* immature survival in HvAV-3h-infected hosts

Several newly-molted-third-instar larvae were exposed to female parasitoids for 24 hours. Then, the larvae were inoculated with HvAV-3h 1, 2, 3, …6, and 7 days later (7 days post-parasitized is a time point when the majority of parasitoid cocoons form). To ensure a manual injection rate, in each day when inoculation conducted, 30 larvae were injected with HvAV-3h as a positive control. In addition, untreated larvae were injected with sterile water, and control larvae were injected with neither the virus nor sterile water. Daily monitoring of the treatments was conducted to check CP. The treated larvae were dissected to examine the development of the parasitoids according to untreated and control groups.

### *Microplitis similis* reproduction with HvAV-3h

Three types of vectors associated with the virus were investigated, the survivors from virus-infected-hosts (5 to 7 DPI), the ones that accompanied with the VI in the first and second experiments, and viruliferous vector. The reproduction was investigated following Tillman *et al.*^21^. The parasitized larvae were reared separately to check IV or CP. The unviruliferous were used as the untreated control to compare life span and reproduction.

Data analysis was performed by SPSS (v16.0). Host infection rates were arcsine square-root-transformed to determine if they met the homoscedasticity assumptions before conducting one-way analysis of variance (ANOVA)^40^.

## Additional Information

**How to cite this article**: Li, S.-J. *et al.* Imperfection works: Survival, transmission and persistence in the system of Heliothis virescens ascovirus 3h (HvAV-3h), *Microplitis similis* and *Spodoptera exigua.* *Sci. Rep.* **6**, 21296; doi: 10.1038/srep21296 (2016).

## Figures and Tables

**Table 1:** Effects of HvAV-3h on reproductive ability of *M. similis.*

Types of females	Replicates (*N*)	Life span (days)	Number of eggs laid in average
Type I	25	6.96 ± 0.23 a	47.16 ± 1.36 a
Type II	29	6.65 ± 0.40 a	45.86 ± 2.23 a
Type III	22	7.36 ± 0.18 a	49.73 ± 1.16 a
Control	25	7.55 ± 0.26 a	46.70 ± 1.31 a

^*^Type I represented females emerged from 5 to 7 days post-parasitism virus-infected hosts; Type II represented females emerged from cocoons of viruliferous females’ offspring; Type III represented viruliferous females. Control group referred to unviruliferous females. Females were provided 10 larvae each day until death to investigate their oviposition performance. Data were shown by means ± S.E.. Different letters indicate significant difference. *P* < 0.05, one-way ANOVA, LSD.
